# Early death during chemotherapy in patients with small-cell lung cancer: derivation of a prognostic index for toxic death and progression

**DOI:** 10.1038/sj.bjc.6690080

**Published:** 1999-02

**Authors:** U N Lassen, K Østerlind, F R Hirsch, B Bergman, P Dombernowsky, H H Hansen

**Affiliations:** Finsen Center, Department of Oncology, National University Hospital, Copenhagen, DK-2100, Denmark; Medical Department F, Hillerød Sygehus, Hillerød, DK-3400, Denmark; Department of Respiratory Medicine, Sahlgrenska Hospital, Gothenburg, SE-413 45, Sweden; Department of Oncology, Herlev University Hospital, Herlev, DK-2730, Denmark

**Keywords:** small-cell lung cancer, chemotherapy, early death, prognostis factors, sepsis

## Abstract

Based on an increased frequency of early death (death within the first treatment cycle) in our two latest randomized trials of combination chemotherapy in small-cell lung cancer (SCLC), we wanted to identify patients at risk of early non-toxic death (ENTD) and early toxic death (ETD). Data were stored in a database and logistic regression analyses were performed to identify predictive factors for early death. During the first cycle, 118 out of 937 patients (12.6%) died. In 38 patients (4%), the cause of death was sepsis. Significant risk factors were age, performance status (PS), lactate dehydrogenase (LDH) and treatment with epipodophyllotoxins and platinum in the first cycle (EP). Risk factors for ENTD were age, PS and LDH. Extensive stage had a hazard ratio of 1.9 (*P* = 0.07). Risk factors for ETD were EP, PS and LDH, whereas age and stage were not. For EP, the hazard ratio was as high as 6.7 (*P* = 0.0001). We introduced a simple prognostic algorithm including performance status, LDH and age. Using a prognostic algorithm to exclude poor-risk patients from trials, we could minimize early death, improve long-term survival and increase the survival differences between different regimens. We suggest that other groups evaluate our algorithm and exclude poor prognosis patients from trials of dose intensification. © 1999 Cancer Research Campaign


British Journal of Cancer (1999) 79(3/4), 515–519
© 1999 Cancer Research Campaign
Article no. bjoc.1998.0080

### Early death during chemotherapy in patients with small-cell lung cancer: derivation of a prognostic index for toxic death and progression

**UN Lassen[1], K Østerlind[2], FR Hirsch[1], B Bergman[3], P Dombernowsky[4] and HH Hansen[1]**

[1]Finsen Center, Department of Oncology, Rigshospitalet, National University Hospital, DK-2100 Copenhagen, Denmark; [2]Medical Department F, Hillerød Sygehus, DK-3400, Hillerød, Denmark; [3]Department of Respiratory Medicine, Sahlgrenska Hospital, SE-413 45 Gothenburg, Sweden; [4]Department of Oncology, Herlev University Hospital, DK-2730 Herlev, Denmark

**Summary** Based on an increased frequency of early death (death within the first treatment cycle) in our two latest randomized trials of combination chemotherapy in small-cell lung cancer (SCLC), we wanted to identify patients at risk of early non-toxic death (ENTD) and early toxic death (ETD). Data were stored in a database and logistic regression analyses were performed to identify predictive factors for early death. During the first cycle, 118 out of 937 patients (12.6%) died. In 38 patients (4%), the cause of death was sepsis. Significant risk factors were age, performance status (PS), lactate dehydrogenase (LDH) and treatment with epipodophyllotoxins and platinum in the first cycle (EP). Risk factors for ENTD were age, PS and LDH. Extensive stage had a hazard ratio of 1.9 ($P = 0.07$). Risk factors for ETD were EP, PS and LDH, whereas age and stage were not. For EP, the hazard ratio was as high as 6.7 ($P = 0.0001$). We introduced a simple prognostic algorithm including performance status, LDH and age. Using a prognostic algorithm to exclude poor-risk patients from trials, we could minimize early death, improve long-term survival and increase the survival differences between different regimens. We suggest that other groups evaluate our algorithm and exclude poor prognosis patients from trials of dose intensification.

**Keywords:** small-cell lung cancer; chemotherapy; early death; prognostis factors; sepsis

Early death (ED), defined as death within the first treatment cycle, was more frequently observed in our two latest randomized trials of combination chemotherapy in unselected patients with small-cell lung cancer (SCLC) compared with our previous trials from the Copenhagen Lung Cancer Group. A total of 12.6% of all patients died during the first treatment cycle, 6.7% in limited disease patients and 18.9% in extensive disease. In our preceding trials, early death was encountered in only 4.2% of all the patients, 3.6% in limited disease and 4.8% in extensive disease.

The increased frequency of early death could be caused by either inferior or more toxic treatment or by an increased number of patients with poor prognosis. However, a recent analysis of our treatment outcome during two decades did not encounter stage migration or change in the distribution of prognostic factors over time (Lassen et al, 1998). According to the International Association for the Study on Lung Cancer (IASLC), toxic death, defined as death during septic episodes, should not exceed 5% (Kristjansen et al, 1990). The risk of early death caused by toxicity, i.e. infections, bleeding episodes etc., may be reduced by identification of high-risk patients. They should primarily be found among those with poor prognostic factors, such as extensive stage of disease, poor performance status (PS), high age and high serum lactate dehydrogenase (LDH). Prompted by the observed increased frequency of early death, the present analysis was carried out focusing on treatment intensity and risk factors for early death, especially early toxic death, in patients with SCLC.

*Received 26 January 1998*
*Revised 13 May 1998*
*Accepted 9 June 1998*

*Correspondence to:* UN Lassen, The Finsen Center, Department of Oncology, National University Hospital, 9 Blegdamsvej, 2100 Copenhagen, Denmark

### PATIENTS AND METHODS

### Treatment intensity

The rates of grade IV leucopenia (< 1000 white blood cells $mm^{-3}$) in the recent trials with a higher rate of early death were compared with the trials conducted from 1976 to 1981. The details of these trials have previously been published (Østerlind et al, 1983, 1986, 1991; Hirsch et al, 1987). Early death was defined as death within the first treatment cycle (4 weeks). Early toxic death (ETD) was defined as early death during episodes with neutropenia and fever, and early non-toxic death (ENTD) was defined as early death in all other instances including progression and concurrent diseases. Patients with neutropenia, but without fever or other signs of infection, were allocated to the ENTD group. Data were obtained from a database of patient records and clinical report forms of trials conducted in our group. A detailed comparison of patient characteristics during this time have recently been performed and published (Lassen et al, 1997).

### Prognostic factors

A detailed analysis of patients included from 1981 to 1991 in our two latest randomized trials was performed. All trials were conducted jointly at Rigshospitalet and Bispebjerg Hospital, Copenhagen, Herlev University Hospital and Hillerød Sygehus, Denmark, and Renströmska Hospital, Gothenburg, Sweden. The

**Table 1** Summary of treatment regimens in trial LU-8109 and LU-8501

| Trial (reference) | Arm | *n* | Regimen |
|---|---|---|---|
| 1981–85 | I | 152 | LCMV alternating with DEV |
| (Pedersen et al, 1987) | II | 153 | Arm I alternating with PvdH |
| | III | 150 | LCVE alternating with LCVP, LCVD, LCVVd, LCVM and LCVH |
| 1985–91 | I | 162 | PTV alternating with arm III |
| (Lassen et al, 1996) | II | 161 | JTV alternating with arm III |
| | III | 159 | CLV alternating with EDV and PVdH |

All regimens administered in 4-week cycles. L, CCNU; C, cyclophosphamide; M, methotrexate; V, vincristine; D, doxorubicin; E, etoposide; P, cisplatin; Vd, vindesine; H, hexamethylmelamine; T, teniposide; J, carboplatin.

studies included a total of 941 consecutive patients, and 937 patients were evaluable for this analysis (Pedersen et al, 1987; Lassen et al, 1996).

There were no limitations for inclusion into the studies with respect to PS, the presence of CNS or bone marrow metastases, liver function tests etc., resulting in a considerably higher fraction of patients with poor prognostic factors. Table 1 describes the study design of the two trials.

All data were stored in a database, and the association between treatment, pretreatment factors and early death was analysed with special reference to toxic deaths. The factors included stage, liver, bone marrow and brain metastases, gender, age, PS and biochemical and haematological variables. Pretreatment characteristics of ETD and ENTD patients were compared with the characteristics of those who survived the first 4 weeks (controls) to identify prognostic factors.

### Statistical analysis

Data were analysed using an interactive program (SPSS, statistical package, 1995). Patient characteristics were compared using $\chi^2$ test for discrete data, and the Kruskal–Wallis test for ordinal scale variables and continuous non-parametric data. Logistic regression analysis was used to analyse factors of prognostic significance for events such as early death, ENTD and ETD. In the analyses, LDH was categorized according to the multiplicity of upper normal levels (450 units $l^{-1}$) and age was dichotomized to <65 or ≥65 years. Survival analysis was calculated by the Kaplan–Meier method, and groups were compared for statistically significant difference using the log-rank method.

### RESULTS

### Treatment comparison

The frequencies of ED and ETD were less than 3.4% and 1.4%, respectively, in our trials performed in 1976–81 (Østerlind et al, 1983, 1986, 1991). In a later randomized trial, these figures changed in a treatment arm with early inclusion of etoposide, based on in vivo cell cyclus analysis. In this arm, ED and ETD were encountered in 19% and 10%, respectively, and grade IV leucopenia was observed in 51% of the patients (Hirsch et al, 1987).

Table 2 shows the distribution of grade IV leucopenia, median white blood cell (WBC) nadir, ENTD and ETD in the different treatment regimens of the trials. The combination of epipodophyllotoxins and platinum (EP) in the induction regimens resulted in an

**Table 2** Numbers of early deaths in relation to treatment regimen

| Treatment arm | ENTD[b] (%) | ETD[c] (%) | Leucopenia grade IV (%) | WBC[a] nadir median ($mm^{-3}$) |
|---|---|---|---|---|
| 1981–85 trial (Pedersen et al, 1987) | | | | |
| I, $n = 152$ | 9 | 1 | 23 | 1700 |
| II, $n = 153$ | 5 | 2 | 29 | 1500 |
| III[a], $n = 150$ | 9 | 9 | 61 | 760[d] |
| 1985–91 trial (Lassen et al, 1996) | | | | 1800 |
| I[a], $n = 162$ | 10 | 6 | 22 | |
| II[a], $n = 161$ | 8 | 5 | 21 | 1600 |
| III, $n = 159$ | 10 | 1 | 37 | 1300 |

ENTD, early non-toxic death; ETD, early toxic death; WBC, white blood cell counts. [a]Epipodophyllotoxins and platinum during first cycle (EP); [b]ENTD, EP vs non-EP, $P$ = n.s.; [c]ETD, EP vs non-EP, $P = 0.0002$; [d]WBC nadir, $P < 0.0001$, Mann–Whitney.

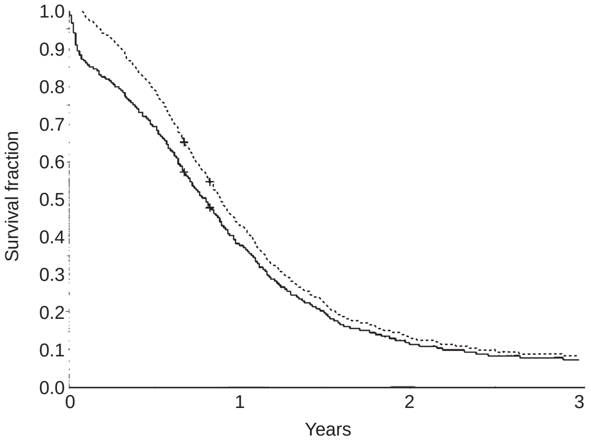

**Figure 1** Kaplan–Meier survival curve of 937 patients with SCLC (—), median survival 309 days (95% CI 289–329), compared with the survival curve when all early deaths, defined as death during the first 4 weeks of chemotherapy, were excluded (– –), median survival 344 days (95% CI 325–363)

increased number of toxic deaths ($P = 0.0002$), whereas treatment had no significant impact on ENTD. The sequential arm in the first trial was more myelotoxic than the other treatment arms (median WBC nadir 760 $mm^{-3}$, $P < 0.001$). In the latest trial, ETD was more frequently observed in the EP regimens even though low median WBC nadir and grade IV leucopenia were more pronounced in the non-EP regimens. The duration of the leucopenic episodes have not been recorded. In general, significantly more ETD occurred among patients receiving EP ($P = 0.04$).

When early death patients were excluded from the two trials, the outcome was significantly changed. In the 1981–85 trial, the survival inferiority of the EP regimens disappeared and no survival difference was present ($P = 0.15$). In the 1985–91 trial, the significant superiority ($P = 0.04$) of the EP regimens increased ($P = 0.002$), as reflected by a difference in median survival of 2 months compared with the previous 1 month. Figure 1 compares the survival curves of all patients, early deaths and the patients surviving the first 4 weeks of treatment.

© Cancer Research Campaign 1999

**Table 3** Pretreatment characteristics for early deaths and controls

| Factor | ENTD *n* = 80 | ETD *n* = 38 | Controls *n* = 819 | Significance |
|---|---|---|---|---|
| Age (median, years) | 65 | 64 | 61 | *P*<0.0005[c] |
| Men/women (%) | 63/37 | 66/34 | 65/36 | NS[b] |
| Stage L/E (%)[a] | 24/76 | 42/58 | 58/42 | *P*<0.0005[b] |
| PS (%) | | | | |
| WHO 0 | 3 | 3 | 23 | *P*<0.0005[c] |
| WHO 1 | 19 | 27 | 44 | |
| WHO 2 | 24 | 27 | 19 | |
| WHO 3 | 33 | 24 | 10 | |
| WHO 4 | 21 | 19 | 4 | |
| AP (median, U $1^{-1}$) | 444 | 293 | 217 | *P*=0.01[c] |
| LDH (median, U $1^{-1}$) | 1030 | 860 | 436 | *P*=0.0005[c] |
| Liver metastases (%) | 38 | 40 | 18 | *P*<0.005[b] |
| Bone marrow metastases (%) | 30 | 35 | 14 | |
| Brain metastases (%) | 9 | 5 | 3 | |

L/E, limited/extensive stage; PS, performance status; ENTD, early non-toxic deaths; ETD, early toxic deaths; AP, alkaline phosphatase; LDH, lactate dehydrogenase. [a]Stage: ENTD vs ETD, *P* = 0.05; [b]chi-squared test; [c]Kruskal–Wallis test.

**Table 4** Logistic regression analysis of factors of prognostic significance for early death (ED), early non-toxic death (ENTD) and early toxic death (ETD)

| | Hazard ratio | 95% CI | Significance |
|---|---|---|---|
| Early death | | | |
| Age | 2.21 | 1.32–3.71 | 0.003 |
| PS | 2.06 | 1.66–2.56 | <0.00005 |
| Stage | 1.15 | 0.65–2.03 | 0.6 |
| LDH | 1.62 | 1.35–1.95 | <0.00005 |
| EP | 2.32 | 1.42–3.79 | 0.0008 |
| Early non-toxic death | | | |
| Age | 2.58 | 1.38–4.83 | 0.003 |
| PS | 1.88 | 1.46–2.42 | <0.00005 |
| Stage | 1.92 | 0.94–3.92 | 0.07 |
| LDH | 1.45 | 1.18–1.78 | 0.0004 |
| EP | 1.15 | 0.66–2.00 | 0.6 |
| Early toxic death | | | |
| Age | 1.36 | 0.65–2.82 | >0.4 |
| PS | 1.86 | 1.34–2.57 | 0.0002 |
| Stage | 0.55 | 0.23–1.32 | 0.2 |
| LDH | 1.59 | 1.20–2.10 | 0.001 |
| EP | 6.66 | 2.66–16.70 | 0.0001 |

PS, performance status (WHO); LDH, lactate dehydrogenase; EP, epipodophyllotoxins and platinum during first cycle; CI, confidence interval.

### Prognostic factors

A total of 941 patients were included in the two trials from 1981 to 1991, and 932 patients were evaluable for the analysis of prognostic factors. One hundred and eighteen patients died during the first 4 weeks after start of treatment (12.6%). The most frequent cause of death was disease progression, but whether a patient died from progression or concurrent diseases could not always be defined because some patients died at home and because autopsy was not performed routinely. A majority of ED occurred during the second week after initiation of chemotherapy when blood cell

**Table 5** Algorithm for predicting risk of early death and poor prognosis

| Characteristic | Score |
|---|---|
| Performance status 3 or 4 | 2 |
| LDH > twice upper normal level | 1 |
| Age ≥65 years | 1 |

Sum score ≥2 allocates patients to risk group. LDH, lactate dehydrogenase; upper normal level 450 U $l^{-1}$.

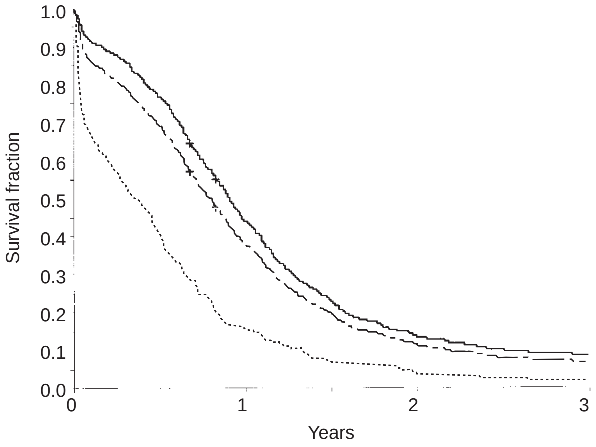

**Figure 2** Kaplan–Meier survival curve of all 937 patients with SCLC (—), median survival 309 days (95% CI 289–329), compared with the favourable prognosis group, *n* = 737 (– - –) median survival 351 days (95% CI 331–371), and the poor prognosis group, defined as patients with PS 3 or 4 or LDH > 900 units $l^{-1}$ and age ≥65 years, *n* = 200 (- - -) median survival 133 days (95% CI 86–180)

counts were at the lowest level, and ETD was recorded in 38 patients (4%). In some instances, ED may have been caused by a combination of toxicity, progression or concurrent diseases. Table 3 shows the pretreatment characteristics of ENTD patients, ETD patients and the control group. Extensive disease and elevated liver enzymes were significantly more frequent among patients who died within the first 4 weeks, whereas the sex ratio was equal between the groups. Also, poor PS was more frequently observed in early deaths. However, 27% of ETD had initial PS 1 and 27% had initial PS 2. The median age and the median values of lactate dehydrogenase (LDH) and alkaline phosphatase (AP) were significantly higher in patients dying early (Kruskal–Wallis). These factors were equally distributed between the ENTD and ETD group, except extensive stage which was less frequently observed with ETD ($P = 0.05$, $\chi^2$ test).

A variable indicating induction regimens containing epipodophyllotoxins and platinum was introduced (EP). This variable was added to the statistically significant variables from the univariate analysis in subsequent logistic regression analyses of factors with prognostic impact on early death, ETD and ENTD (Table 4). Significant risk factors of early death were high age, poor performance status, elevated LDH and EP treatment. High age, poor performance status and elevated LDH were statistically significant risk factors for ENTD. Extensive stage of disease resulted in a hazard ratio of 1.9, but the confidence interval did not reach significance ($P = 0.07$). EP had no influence on ENTD, and age was not a significant factor in a separate analysis of limited

*© Cancer Research Campaign 1999*

disease only. The same analysis of ETD showed that poor PS and elevated LDH were statistically significant. EP was a powerful factor of ETD with a hazard ratio of 6.7. Neither bone marrow nor liver or brain metastases were independent predictive factors for early death or early toxic death. The patients with PS 1 or 2 had significantly increased hazard ratios for elevated LDH, age ≥65 and EP regimens (hazard ratios 1.7, 2.4 and 5.3 respectively). Based on hazards from the analyses of both ENTD and ETD, a prognostic algorithm for early death was introduced. The major prognostic factors were weighted according to the hazard. PS 3 or 4 scored 2 points, LDH > twice upper normal level and age >65 years each scored 1 point. Stage was not a significant prognostic factor for ED and was not included in the algorithm. Patients were allocated to the poor prognosis group if the total score was 2 points or more (Table 5). The poor prognosis group included patients with PS 3 or 4 or LDH > twice upper normal level and age ≥65. With this algorithm, a majority of EDs could be excluded; this group accounted for 21% of the patients. The remaining 79% of the patients were allocated to a favourable prognosis group and a survival analysis was performed. In the trial from 1981 to 1985, the inferiority of the EP regimen was reduced after exclusion of the majority of early deaths, but it was still significant ($P = 0.03$). In the 1985–91 trial, the superiority of EP compared with non-EP regimens became more pronounced when the analysis only included the favourable prognosis patients (median survival 376 days vs 310 days respectively, $P = 0.03$). In the favourable group, 3-year survival was 11.4% compared with 7.8% among all patients receiving EP. These changes were comparable to the changes resulting when early deaths were excluded from the survival analyses. Figure 2 compares the survival curves for all patients, the favourable and the poor prognosis groups.

In the poor prognosis group, median survival was 133 days, and 2- and 3-year survival rates were 4.5% and 2.0% respectively. In this group of patients, the frequency of early death was 33%, and among the patients receiving EP the frequency was 41%.

### DISCUSSION

Previous studies of early death have identified stage, PS and liver enzymes as significant prognostic factors, but also epipodophyllotoxins have been proposed to be responsible for an increased risk of early toxic death (Morittu et al, 1989). It has been suggested that this possibly could be caused by an association between hepatic metastases, compromised liver function and elimination of etoposide (Pfluger et al, 1987; Morittu et al, 1989). However, a discrimination between early death due to progression and early death due to infection was not made in these studies. Other studies have focused on toxic death alone. Radford et al (1993) identified factors predicting septic complications in an analysis of 382 patients and derived a prognostic index including PS ≥3, age >50 and three or more drugs. However, the Radford et al (1993) study did not discriminate between early or later toxicity. Another large analysis of 2196 patients modified the Radford index by omitting age and including WBC ≥10 000 $mm^{-3}$. In addition, PS was modified to ≥2 and the number of drugs increased to four or more. Patients dying from sepsis were not recorded and the analysis was exclusively based on excess death rates during the first treatment cycle (Stephens et al, 1994).

The fact that ETD was more frequent in the EP regimens in our latest trial despite the fact that leucopenia was more pronounced in the control regimen indicates the complexity of this problem. The schedule of administration of epipodophyllotoxins has previously been shown to be important for outcome. Based on in vivo cell kinetic observations, it has been shown that etoposide has maximal activity against cells in the S and $G_2$ phases (Kalwinsky et al, 1983). In most regimens, epipodophyllotoxins, therefore, are administered for 3–5 days early in each cycle. This was also the case in our regimens. This could possibly result in nadir periods of longer duration compared with regimens in which the drugs are administered in 1 day. Unfortunately, our haematological surveillance did not include daily blood cell counts. This was only the case when grade IV toxicity had resulted in hospitalization of the patients. Therefore, a comparison of nadir durations among the different regimens could not be performed.

Risk of early death due to infection seems to be more independent of stage, and fatal toxicity occurred in both stages. Increased LDH was an independent prognostic factor, presumably related to tumour burden rather than to hepatic function. PS was found to be the most powerful risk factor of both toxic and non-toxic early death. Stratification for PS and LDH is extremely important in both disease stages because these patients have an increased risk of early toxic death when treated with extraordinary myelotoxic combination chemotherapy. It is noteworthy that more than 50% of ETD occurred among patients with PS 1 or 2. A majority of these patients had increased LDH and were more than 65 years old. Therefore, it is difficult to propose a simple algorithm predicting risk of ETD. Patients with PS 1 in general have a favourable prognosis and should be included in clinical trials. Our data suggest that good performance patients should receive increased haematological surveillance if they are more than 65 years old or have elevated LDH. In addition, treatment with colony-stimulating factors could possibly reduce the risk of ETD in this group, but this needs further evaluation in prospective clinical trials. If the patients are older than or equal to 65 years and have moderately elevated LDH, they should be excluded from trials with potent myelosuppressive agents.

Most phase II and III trials of high-dose chemotherapy and dose intensification have been disappointing with regard to survival (Stahel et al, 1984; Johnson et al, 1987; Jackson et al, 1988; Klasa et al, 1991; Katakami et al, 1996; Fetscher et al, 1997; Murray et al, 1997). Most of these trials only included patients with good performance status and thereby excluded approximately 15% of the patients. If patients with other poor prognostic factors had been excluded, the results might have been in favour of treatment intensification. Epipodophyllotoxins can safety be administered to elderly patients (Bork et al, 1991, 1997). The risk of toxic death is correlated to dose intensity and scheduling with other agents. According to this and other similar analyses, the goal for treatment of patients with poor prognosis primarily is palliative. It should not be too intensive (Girling et al, 1996*a*), albeit monotherapy with etoposide may be suboptimal (Girling et al, 1996*b*; Souhami et al, 1997) compared with intravenous combination chemotherapy. Our analysis did not address how to treat such patients; epipodophyllotoxins and platinum are widely used, and dosage and scheduling will always depend on a narrow balance between efficacy and toxicity.

Our algorithm included predictive factors for both ENTD and ETD. The frequency of early death among the excluded group of patients was as high as 41% when receiving EP. We propose that other groups use and evaluate our algorithm to exclude patients

 *© Cancer Research Campaign 1999*

with poor prognosis from regimens with potent myelosuppressive agents and trials with dose intensification or dose escalation. Exclusion of such patients may reveal that dose intensification will result in a significantly improved outcome. This should encourage further randomized studies in SCLC in patients with a favourable prognosis. These account for approximately 80% of the patients who better tolerate intensive treatment, and an increased toxicity is acceptable provided more patients become long-term survivors.

### ACKNOWLEDGEMENT

This study was supported by The Foundation For The Support Of Medical Cancer Treatment.

### REFERENCES

Bork E, Ersbøll J, Dombernowsky P, Bergman B, Hansen M and Hansen HH (1991) Teniposide and etoposide in previously untreated small-cell lung cancer: a randomised study. *J Clin Oncol* **9**: 1627–1631

Bork E, Hirsch FR, Jeppesen N, Lassen U, Vallentin S, Østerlind K, Mejer J, Ingeberg S, Bergman B and Dombernowsky P (1997) Oral etoposide (VP-16) every 3 wks. or continuously to elderly patients with small cell lung cancer (SCLC). Preliminary results of a randomaized study. *Lung Cancer* **18** (suppl. 1): 25

Fetscher M, Brugger W, Engelhardt R, Kanz L, Hasse J, Frommhold H, Wenger M, Lange W and Mertelsmann R (1997) Dose-intense therapy with etoposide, ifosfamide, cisplatin, and epirubicin (VIP-E) in 100 consecutive patients with limited- and extensive-disease small-cell lung cancer. *Ann Oncol* **8**: 49–56

Girling DJ, Hopwood P, Lallemand G, Machin D, Stephens RJ and Baily AJ (1996*a*) Randomised trial of four-drug vs. less intensive two-drug chemotherapy in the palliative treatment of patients with small-cell lung cancer (SCLC) and poor prognosis. *Br J Cancer* **73**: 406–413

Girling D, Thatcher N, Clark PI, Hopwood P, Twiddy S and Stephens RJ (1996*b*) Comparison of oral etoposide and standard intravenous multidrug chemotherapy for small-cell lung cancer: a stopped multicentre randomised trial. Medical Research Council Lung Cancer Working Group. *Lancet* **31**: 563–566

Hirsch FR, Hansen HH, Hansen M, Østerlind K, Vindeløv LL, Dombernowsky P and Sörensson S (1987) The superiority of combination chemotherapy including etoposide based on in vivo small-cell lung cancer: a randomized trial of 288 consecutive patients. *J Clin Oncol* **5**: 585–591

Jackson DV, Case LD, Zekan PJ, Powell BL, Caldwell RD, Bearden JD, Nelson EC, Muss AB, Cooper MR, Richard S II F, White DR, Cruz JM, Caponera ME, Furr CS, Spurr CL and Capizzi RL (1988) Improvement of long-term survival in extensive small-cell lung cancer. *J Clin Oncol* **6**: 1161–1169

Johnson DH, Einhorn LH, Birch R, Vollmer R, Perez C, Krauss S, Omura G and Greco A (1987) A randomized comparison of high-dose versus conventional-dose cyclophosphamide, doxorubicin, and vincristine for extensive-stage small cell lung cancer: a phase III trial of the Southeastern Cancer Study Group. *J Clin Oncol* **5**: 1731–1738

Kalwinsky DK, Look AT, Ducore J and Fridland AL (1983) Effects of the epipodophyllotoxin VP-16-213 on the cell cycle traverse, DNA synthesis, and DNA strand size in cultures of human leukemic lymphoblasts. *Cancer Res* **43**: 1592–1597

Katakami N, Takada M, Negore S, Ota K, Fujita J, Aryoshi Y, Ikegami H and Fukuoka M (1996) Dose escalation study of carboplatin with fixed-dose etoposide plus granulocyte-colony stimulating factor in patients with small cell lung carcinoma. *Cancer* **77**: 63–70

Klasa RJ, Murray N and Coldman AJ (1991) Dose-intensity meta-analysis of chemotherapy regiments in small-cell carcinoma of the lung. *J Clin Oncol* **9**: 499–508

Kristjansen PEG and Hansen HH (1990) Management of small cell lung cancer: a summary of the third International Association for the Study of Lung Cancer Workshop on Small Cell Lung Cancer. *J Natl Cancer Inst* **82**: 263–266

Lassen U, Kristjansen PEG, Østerlind K, Bergman B, Sigsgaard TC, Hirsch FR, Hansen M, Dombernowsky P and Hansen HH (1996) Superiority of cisplatin or carboplatin in combination with teniposide and vincristine in the induction chemotherapy of small-cell lung cancer. A randomized trial with 5 years follow up. *Ann Oncol* **7**: 365–371

Lassen U, Hirsch FR, Østerlind K, Bergman B, Dombernowsky P and Hansen HH (1998) Outcome of combination chemotherapy in extensive stage small cell lung cancer. Any treatment related progress? *Lung Cancer* **20**: 151–160

Morittu L, Earl HM, Souhami RL, Ash CM, Tobias JC, Geddes DM, Harper PG and Spiro SG (1989) Patients at risk of chemotherapy-associated toxicity in small cell lung cancer. *Br J Cancer* **59**: 801–804

Murray N, Livingston RB, Shephard FA, James K, Zee BC, Langle-Ben A, Kraut M, Bearden J, Goodwin J, Crafton C, Turrisi AT, Walde D, Croft H and Ottaway J (1997) A randomized study of CODE plus thoracic irradiation versus alternating CAV/EP for extensive stage small cell lung cancer (ESCLC). *Proc Am Soc Clin Oncol* **16**: 456

Østerlind K, Sörensson S, Hansen HH, Dombernowsky P, Hirsch FR, Hansen M and Rørth M (1983) Continuous versus alternating combination chemotherapy for advanced small cell carcinoma of the lung. *Cancer Res* **43**: 6085–6089

Østerlind K, Hansen HH, Hansen HS, Dombernowsky P, Hansen M and Rørth M (1986) Chemotherapy versus chemotherapy plus irradiation in limited small cell lung cancer. Results of a controlled trial with 5 years follow-up. *Br J Cancer* **54**: 7–17

Østerlind K, Hansen M, Hirsch FR, Dombernowsky P, Sörensson S, Pedersen AG and Hansen HH (1991) Combination chemotherapy of limited small cell lung cancer. A controlled trial on 222 patients comparing two alternating regimens. *Ann Oncol* **2**: 41–46

Pedersen AG, Østerlind K, Vindeløv L, Sörensson S, Hansen M, Dombernowsky P and Hansen HH (1987) Alternating or continuous chemotherapy of small cell lung cancer. A three armed randomized trial (abstract). *Proc Am Soc Clin Oncol* **6**: 187

Pfluger K-H, Schmidt L, Merkel M, Jungclas H and Havemann K (1987) Drug monitoring of etoposide (VP16-213). Correlation of pharmacokinetic parameters to clinical and biochemical data from patients receiving etoposide. *Cancer Chemother Pharmacol* **20**: 59

Radford JA, Ryder WDJ, Dodwell D, Anderson H and Thatcher N (1993) Predicting septic complications of chemotherapy: an analysis of 382 patients treated for small cell lung cancer without dose reduction after major sepsis. *Eur J Cancer* **29A**: 81–86

Souhami RL, Spiro SG, Rudd RM, Ruiz De Elvera MC, James LE, Gower NH, Lamont A and Harper PG (1997) Five-day oral etoposide treatment for advanced small-cell lung cancer. Randomized comparison with intravenous chemotherapy. *J Natl Cancer Inst* **89**: 577–580

SPSS for Windows (1995) Statistical package, version 6.1.3

Stahel RA, Takvorian RW, Skarin AT and Canellos GP (1984) Autologous bone marrow transplantation following high-dose chemotherapy with cyclophosphamide, BCNU, and VP-16 in small cell carcinoma of the lung and a review of the literature. *Eur J Cancer Clin Oncol* **20**: 1233–1238

Stephens RJ, Girling DJ and Machin D (1994) Treatment related deaths in small cell lung cancer trials: can patients at risk be identified? *Lung Cancer* **11**: 259–274

© Cancer Research Campaign 1999

## References

[PDF_00430] Bork E., Ersbøll J., Dombernowsky P., Bergman B., Hansen M., Hansen H. H. (1991). Teniposide and etoposide in previously untreated small-cell lung cancer: a randomized study.. J Clin Oncol.

[PDF_00457] Jackson D. V., Case L. D., Zekan P. J., Powell B. L., Caldwell R. D., Bearden J. D., Nelson E. C., Muss H. B., Cooper M. R., Richards F. (1988). Improvement of long-term survival in extensive small-cell lung cancer.. J Clin Oncol.

[PDF_00459] Johnson D. H., Einhorn L. H., Birch R., Vollmer R., Perez C., Krauss S., Omura G., Greco F. A. (1987). A randomized comparison of high-dose versus conventional-dose cyclophosphamide, doxorubicin, and vincristine for extensive-stage small-cell lung cancer: a phase III trial of the Southeastern Cancer Study Group.. J Clin Oncol.

[PDF_00464] Kalwinsky D. K., Look A. T., Ducore J., Fridland A. (1983). Effects of the epipodophyllotoxin VP-16-213 on cell cycle traverse, DNA synthesis, and DNA strand size in cultures of human leukemic lymphoblasts.. Cancer Res.

[PDF_00472] Klasa R. J., Murray N., Coldman A. J. (1991). Dose-intensity meta-analysis of chemotherapy regimens in small-cell carcinoma of the lung.. J Clin Oncol.

[PDF_00475] Kristjansen P. E., Hansen H. H. (1990). Management of small cell lung cancer: a summary of the Third International Association for the Study of Lung Cancer Workshop on Small Cell Lung Cancer.. J Natl Cancer Inst.

[PDF_00480] Lassen U., Kristjansen P. E., Osterlind K., Bergman B., Sigsgaard T. C., Hirsch F. R., Hansen M., Dombernowsky P., Hansen H. H. (1996). Superiority of cisplatin or carboplatin in combination with teniposide and vincristine in the induction chemotherapy of small-cell lung cancer. A randomized trial with 5 years follow up.. Ann Oncol.

[PDF_00487] Morittu L., Earl H. M., Souhami R. L., Ash C. M., Tobias J. S., Geddes D. M., Harper P. G., Spiro S. G. (1989). Patients at risk of chemotherapy-associated toxicity in small cell lung cancer.. Br J Cancer.

[PDF_00496] Osterlind K., Sörenson S., Hansen H. H., Dombernowsky P., Hirsch F. R., Hansen M., Rørth M. (1983). Continuous versus alternating combination chemotherapy for advanced small cell carcinoma of the lung.. Cancer Res.

[PDF_00510] Pflüger K. H., Schmidt L., Merkel M., Jungclas H., Havemann K. (1987). Drug monitoring of etoposide (VP16-213). Correlation of pharmacokinetic parameters to clinical and biochemical data from patients receiving etoposide.. Cancer Chemother Pharmacol.

[PDF_00442] (1996). Randomised trial of four-drug vs less intensive two-drug chemotherapy in the palliative treatment of patients with small-cell lung cancer (SCLC) and poor prognosis. Medical Research Council Lung Cancer Working Party.. Br J Cancer.

[PDF_00518] Souhami R. L., Spiro S. G., Rudd R. M., Ruiz de Elvira M. C., James L. E., Gower N. H., Lamont A., Harper P. G. (1997). Five-day oral etoposide treatment for advanced small-cell lung cancer: randomized comparison with intravenous chemotherapy.. J Natl Cancer Inst.

[PDF_00523] Stahel R. A., Takvorian R. W., Skarin A. T., Canellos G. P. (1984). Autologous bone marrow transplantation following high-dose chemotherapy with cyclophosphamide, BCNU and VP-16 in small cell carcinoma of the lung and a review of current literature.. Eur J Cancer Clin Oncol.

[PDF_00527] Stephens R. J., Girling D. J., Machin D. (1994). Treatment-related deaths in small cell lung cancer trials: can patients at risk be identified? Medical Research Council Lung Cancer Working Party.. Lung Cancer.

